# Inhibitor analysis revealed that clathrin-mediated endocytosis is involed in cellular entry of type III grass carp reovirus

**DOI:** 10.1186/s12985-018-0993-8

**Published:** 2018-05-24

**Authors:** Hao Wang, Weisha Liu, Meng Sun, Dubo Chen, Lingbing Zeng, Liqun Lu, Jing Xie

**Affiliations:** 1 National Pathogen Collection Center for Aquatic Animals, Shanghai Ocean University, Shanghai, People’s Republic of China; 2 Key Laboratory of Freshwater Aquatic Genetic Resources, Ministry of Agriculture and Rural Affairs, Shanghai, People’s Republic of China; 3 National Demonstration Center for Experimental Fisheries Science Education, Shanghai Ocean University, Shanghai, People’s Republic of China; 4 Department of Laboratory Medicine, the frist affiliated hospital of Sun Yat-sen University, Guangzhou, People’s Republic of China; 5 Division of Fish Disease, Yangtze River Fisheries Research Institute, Chinese Academy of Fishery Sciences, Wuhan, Hubei People’s Republic of China; 6 Shanghai Engineering Research Center of Aquatic Product Processing & Preservation, Shanghai, People’s Republic of China

**Keywords:** *Ctenopharyngodon idellus*, GCRV104, Ammonium chloride, Clathrin, *Sedoreovirinae*

## Abstract

**Background:**

Grass carp (*Ctenopharyngodon idella*) hemorrhagic disease is caused by an acute infection with grass carp reovirus (GCRV). The frequent outbreaks of this disease have suppressed development of the grass carp farming industry. GCRV104, the representative strain of genotype III grass carp (*Ctenopharyngodon idella*) reovirus, belongs to the *Spinareovirinae* subfamily and serves as a model for studying the strain of GCRV which encodes an outer-fiber protein. There is no commercially available vaccine for this genotype of GCRV. Therefore, the discovery of new inhibitors for genotype III of GCRV will be clinically beneficial. In addition, the mechanism of GCRV with fiber entry into cells remains poorly understood.

**Methods:**

Viral entry was determined by a combination of specific pharmacological inhibitors, transmission electron microscopy, and real-time quantitative PCR.

**Results:**

Our results demonstrate that both GCRV-JX01 (genotype I) and GCRV104 (genotype III) of GCRV propagated in the grass carp kidney cell line (CIK) with a typical cytopathic effect (CPE). However, GCRV104 replicated slower than GCRV-JX01 in CIK cells. The titer of GCRV-JX01 was 1000 times higher than GCRV104 at 24 h post-infection. We reveal that ammonium chloride, dynasore, pistop2, chlorpromazine, and rottlerin inhibit viral entrance and infection, but not nystatin, methyl-β-cyclodextrin, IPA-3, amiloride, bafilomycin A1, nocodazole, and latrunculin B. Furthermore, GCRV104 and GCRV-JX01 infection of CIK cells depended on dynamin and the acidification of the endosome. This was evident by the significant inhibition following prophylactic treatment with the lysosomotropic drug ammonium chloride or dynasore.

**Conclusions:**

Taken together, our data have suggested that GCRV104 enters CIK cells through clathrin-mediated endocytosis in a pH-dependent manner. We also suggest that dynamin is critical for efficient viral entry. Additionally, the phosphatidylinositol 3-kinase inhibitor wortmannin and the protein kinase C inhibitor rottlerin block GCRV104 cell entry and replication.

## Background

Grass carp *Ctenopharyngodon idella* reovirus (GCRV), also known as grass carp hemorrhage virus, is a pathogenic virus isolated from grass carp hemorrhagic disease. This disease negatively affects grass carp production in Asian countries, especially China [1]. The clinical symptoms of infection are hemorrhages in organs, showing spots or plate forms, in combination with some or all of the following symptoms: exophthalmia, body darkening, hemorrhage of the mouth cavity, hemorrhagic or pale gills, gill-rot, red-skin, and hemorrhage at the base of fins and gill covers [2]. GCRV belongs to the genus *Aquareovirus,* of family *Reoviridae* [3]. Over the last decade, many isolates of GCRV have been reported, and several isolates have been completely sequenced, such as GCRV-873 [4], GCRV-HZ08 [5], HGDRV (formerly GCRV-104) [6], GCRV-JX01 [7], GCRV-JX02 [7], and GCRV-AH528 [8]. The *Reoviridae* family is the largest of the eight recognized double-stranded RNA (dsRNA) virus families [9]. Members of *Reoviridae* are further divided into two subfamilies, the *Sedoreovirinae* and the *Spinareovirinae,* based on their virus capsid structure [9]. The virus strains of *Spinareovirinae* are turreted reoviruses, which have large spikes, or turrets, situated on the virus core structure, while the *Sedoreovirinae* are non-turreted [6]. According to phylogenetic relationship between GCRV isolates, Max L. et al. [10, 11] have demonstrated that the isolates of GCRV can be divided into three genotypes, with representative isolates genotype I (GCRV-873, GCRV-JX01), genotype II (GCRV-HZ08, GCRV106), and genotype III (GCRV104). As the typical strain of Aquareovirus C, genotype I GCRV (GCRV-873, GCRV-JX01) has been investigated extensively due to its strong virulence both in vivo and in vitro [1]. It encodes five nonstructural proteins (NS80, NS38, NS31, NS26, and NS16) and seven structural proteins (VP1-VP7), with no outer fiber protein (spike protein) [12]. In contrast to genotype I GCRV (GCRV-873, GCRV-JX01), genotype II (GCRV-HZ08, GCRV106) and genotype III (GCRV104) of GCRV possess an outer fiber, or NS-FAST protein [10]. Currently, treating GCRV infection remains difficult; although, a live vaccine [13] was developed for the GCRV-892 isolates and is widely used in China. Still, there are no effective therapies against multiple genotypes of GCRV infection to date. In addition, there is little known on the preventive and therapeutic strategies against genotype III (GCRV104) of GCRV.

Fang Qin. et al. [3] demonstrated a well-orchestrated process for nonenveloped virus entry involving autocleavage of the penetration protein prior to exposure of its membrane-insertion finger. Many pathways have been reported for virus entry, such as receptor-mediated endocytosis followed by pH-dependent or -independent fusion from endocytic compartments, or even pH-independent fusion at the plasma membrane coupled with receptor-mediated signaling and coordinated disassembly of the actin cortex [14]. Furthermore [15], clathrin-mediated [16], caveolar-mediated [17], micropinocytosis [18], and clathrin/caveolae-independent endocytosis pathway are utilized by many viruses. However, little is known on the mechanism of entry of the GCRV strains of *Spinareovirinae,* particularly genotype III (GCRV104). Currently, many studies in virus entry focus on the use of inhibitors [19]. In this report, we investigate candidate inhibitors for genotype III grass carp reovirus (GCRV104) entry and infection.

## Methods

### Cells and viruses

Grass carp (*Ctenopharyngodon idellus*) kidney cells (CIK) [7] were grown at 28 °C in M199 (Gibco BRL, USA) media with 50 U/ml of penicillin, 50 mg/ml streptomycin, and 10% fetal calf serum (Biosource, Gibco BRL, USA). The viral strain GCRV-JX01 was isolated and preserved in our laboratory [7]. GCRV-104 (HGDRV) (CCTCC NO: V201217) strain was isolated from Yangtze River fisheries research institute [6]. The viral stocks were prepared by passage in CIK cells and purified as previously described [7]. GCRV particles were extracted by differential centrifugation from the collected supernatant: CIK cell fragments were removed at 8500 x g for 30 min at 4 °C, then, the GCRV particles were concentrated at 80,000 x g for 3 h at 4 °C [20].

### Inhibitors

The pharmacological inhibitors’ concentration used in our study is based on previous research (Table 1) [21, 22]. Inhibitors were prepared as follows: pistop2, dynasore, rottlerin, nystatin, wortmannin, bafilomycin A1, Latrunculin B, nocodazole, IPA-3, and amiloride were purchased from ApexBio (Houston, USA). Chlorpromazine (CPZ) was purchased from Selleck. Stock solutions of inhibitors were dissolved in dimethyl sulfoxide (DMSO), except Latrunculin B which was dissolved in ethanol according to the manufacturer’s instruction. Ammonium chloride (NH_4_Cl) and methyl-β-cyclodextrin were bought from Sigma and dissolved in H_2_O.

**Table 1:** Variables from the questionnaire of the HBSC study analyzed in this study

Inhibitor	Target	Highest concentration
Pistop2	CME	5 μM (GCRV 104); 25 μM (GCRV JX01)
CPZ	CME	10 μM (GCRV 104; GCRV JX01)
Nystatin	Caveola-dependent endocytosis	15 μM (GCRV 104)
Methyl-β-cyclodextrin	Caveola-dependent endocytosis	1 mM (GCRV 104)
Dynasore	Dynamin	10 μM (GCRV 104)
Wortmannin	PI3K	5 μM (GCRV 104)
IPA-3	Pak1	20 μM (GCRV 104)
Rottlerin	PKC	10 μM (GCRV 104)
NH_4_Cl	PH	20 mM (GCRV 104)
Amiloride	NHE	10 μM (GCRV 104)
Bafilomycin A1	Vacuolar ATPas inhibition	2 nM (GCRV 104)
Nocodazole	Microtubules	10 μM (GCRV 104)
Latrunculin B	Actin microfilaments	0.5 μM (GCRV 104)

### Effect of inhibitors on GCRV entry and infection

CIK cells were seeded in 12-well plates and pretreated with indicated inhibitors or fresh medium without inhibitors (as a control group) for 1 h at 4 °C. For virus infection analysis, cells were infected with GCRV104 or GCRVJX01 (MOI = 5) for 30 min adsorption at 4 °C. Cultures were then quickly warmed to 28 °C for another 1 h to allow viral internalization. Next, non-internalized viruses were removed with two PBS washes. The supernatant was collected for analysis after 5 days (GCRV104) or 24 h (GCRVJX01). All inhibitors were present throughout the experiment. For virus entry analysis, cells were then adsorbed with virus (MOI = 50) for 30 min at 4 °C. Next, cells were transferred to 28 °C for 30 min. The medium containing inhibitors was added once again after removal of non-internalized viruses. The cells were collected in TRIzol reagent (Invitrogen) after 24 h (GCRV104) or 12 h (GCRVJX01). Total RNA was extracted from CIK cells and virus entry into host cells was analyzed by RT-PCR.

### Quantitative real-time RT-PCR assay

The quantitative real-time RT-PCR assay was employed to detect virus infection in CIK cells. cDNAs of purified RNAs, from infected cells or supernatants, were synthesized using PrimeScript reverse transcription system (TakaRa, Japan) following its product protocol. Real-time RT-PCR was performed according to our previous methods [7, 23] using a CFX96 real-time PCR system (Bio-rad, USA) with the primer pairs of JX01F: CAAGACCATTCAAGACTC; JX01R: TCACTCACTTCGACTAAT and 104F: ATCGTCTTCAACCGCATAG; 104R: GGGCGTTACTTCCCTCAAC.

### Western blot

CIK cells were treated with inhibitors and virus as described above. For western blot analysis, cells were collected and lysed in SDS-PAGE loading buffer (Beyotime Institute of Biotechnology, China). Samples were separated by electrophoresis on a 10% polyacrylamide gel and transferred to 0.45 μm Immuno-Blot Polyvinylidene fluoride (PVDF) membrane (Merck Millipore, Darmstadt, Germany). Membranes were then blocked in 5% skim milk at room temperature for 1 h. The separated proteins were then incubated with a homemade polyclonal antibody for GCRV104-VP4 (1:2000) or GCRVJX01-VP7 (1:2000) at 4 °C overnight. GAPDH was detected simultaneously as an internal control. Goat anti-mouse IgG HRP (1:4000) or goat anti-rabbit IgG HRP (1:4000) were used as secondary antibodies, at room temperature for 1 h. The signal was developed by ECL Plus western blot analysis kit (Amersham Pharmacia Biotech, Taiwan, China).

### Electron microscopy

CIK cells were cultured in dish (Thermo Fisher) to 80% confluency. Cells were pretreated with indicated inhibitors for 1 h at 4 °C, and then adsorbed with virus (MOI = 50) for 30 min at 4 °C. Cells were then placed at 28 °C for 30 min. Following 3 PBS washes, cells were treated with inhibitors again for 48 h at 28 °C. The processed cells were then fixed at 4 °C in electron microscopy grade fixative. All sample grids were examined under a transmission electron microscope (Hitachi 7000-FA) as previously described [24].

### Statistical analysis

Error bars indicate the standard errors of the means. To measure the effect of the inhibitors on GCRV-104 infection, mean values for a minimum of triplicate samples were compared using paired (normalized) Student’s *t*-tests. A value of *p* < 0.05 was considered to be statistically significant using GraphPad statistics software.

## Results

### Infection and entry kinetics of GCRV104 and GCRV JX01

**Fig. 1:**
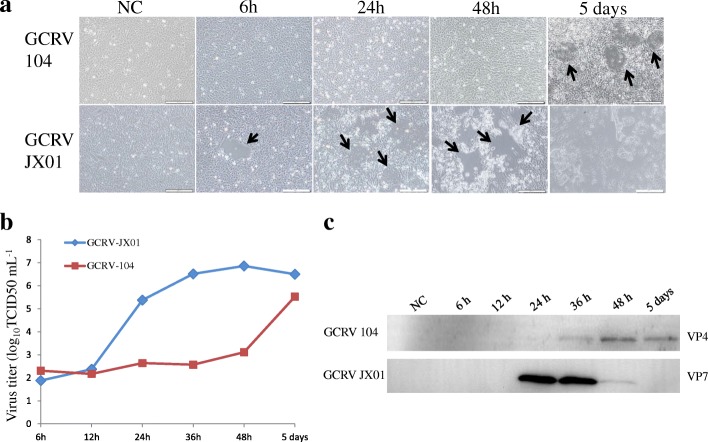
Infection kinetics of GCRV104 and GCRV-JX01 in CIK cells. **a**. CPE of GCRV104 and GCRV-JX01 infection viewed under a visible light phase microscope. The arrow indicates cytopathic effect (CPE). **b** TCID_50_ assay of virus yield in supernatants. **c** Western blot analysis for viral replication level at different time points

The typical CPE of GCRV104 was observed at 5 days post-infection. However, for GCRV JX01 infected CIK cells, CPE appeared as early as 6 h post-infection. The CPE is exacerbated as the infection progresses, with the cell monolayer completely destroyed by day 5 post infection (Fig. 1a).

We investigated the replication kinetics of different GCRV genotypes by TCID_50_ assay of harvested supernatants. GCRV104 replicated slower than GCRV JX01 in CIK cells. The titer of GCRV JX01 was 1000 times higher than GCRV104 at 24 h post-infection (Fig. 1b).

The entry kinetics of GCRV104 and GCRV JX01 were demonstrated by western blot. The two types of GCRV had different protein expression levels at different time points. As shown in Fig. 1c, GCRV104 could be detected in CIK cells at 36 h post-infection, but the GCRV JX01 could be detected earlier, at 24 h post-infection (Fig. 1c). This is most likely explained by the earlier entry of GCRV JX01 into host cells than GCRV104.

### GCRV104 entry is dependent on dynamin in low pH environment

**Fig. 2:**
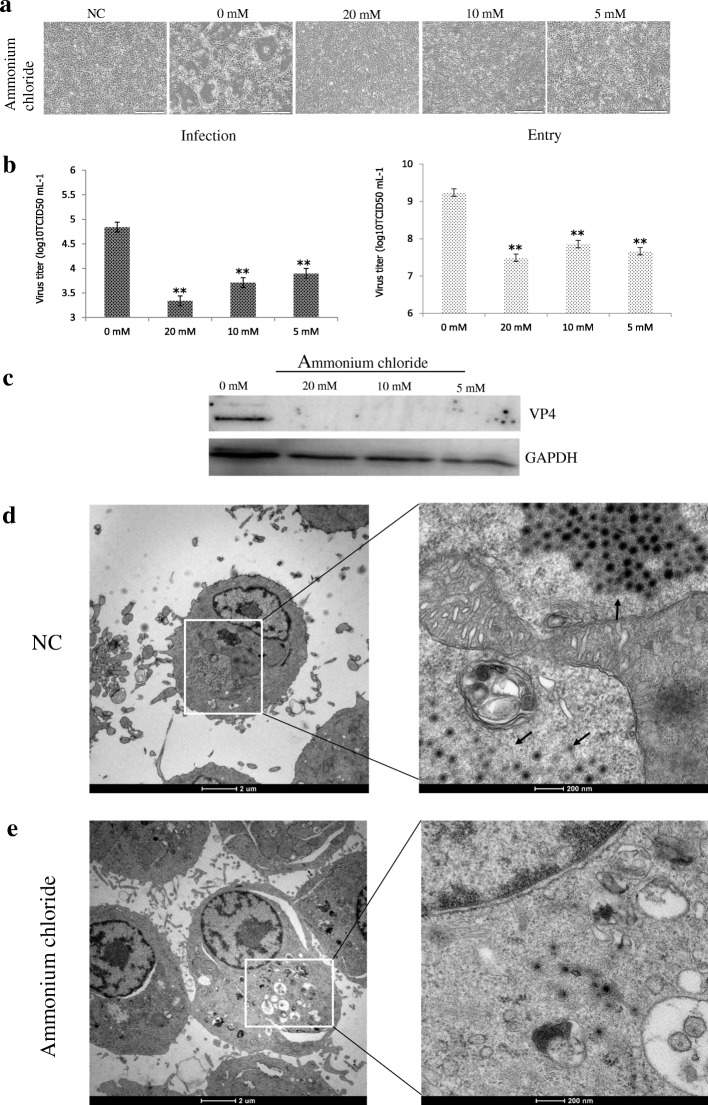
Effect of ammonium chloride on the production of progeny virus. CIK cells were treated with ammonium chloride at the indicated concentrations. **a** CPE of GCRV104 infection viewed under a visible light phase microscope. **b** Rt-PCR assay of virus yield in the supernatants (infection) and CIK cells (entry). (unpaired t-test, * *P* < 0.05 and ** *P* < 0.01). **c** Western blot analysis to monitor viral replication level in CIK cells. **d** and **e** Ultrastructural visualization of the uptake of GCRV into CIK cells by transmission electron microscopy. Cells were pretreated with indicated inhibitors (**d**: PBS, **e**: ammonium chloride, 20 mM) for 1 h at 4 °C, then adsorbed with virus (MOI = 50) for 30 min at 4 °C. The cultures were quickly warmed to 28 °C to start infection, and uninternalized virions were removed at 0.5 hpi. After PBS washes, cells were treated with inhibitors again for 48 h at 28 °C. The processed cells were then fixed in electron microscopy grade fixative at 4 °C

The CPE in cells treated with various concentrations of NH_4_Cl at 5 d post-infection was quantified. Virally-induced typical CPE was observed in varying NH_4_Cl concentrations compared to control (Fig. 2a). The viral infection and entry in CIK cells treated with NH_4_Cl was significantly lower than that of the control group (*P* < 0.01) (Fig. 2b). This data indicates that NH_4_Cl inhibits GCRV104 infection and entry. Furthermore, western blots determined that viral protein vp4 was reduced in GCRV104 infected cells treated with NH_4_Cl (Fig. 2c). These studies suggest that the entry of GCRV104 is dependent on a low pH. To further confirm this data, we visualized the infection of GCRV104 by transmission electron microscopy (TEM). As shown in Fig. 2 D and E, electron micrographs of infected CIK cells with GCRV104 reveal large numbers of virus particles (indicated by arrows), many vacuolated mitochondria, and viral inclusion bodies (indicated by arrows). Compared to the negative group, little GCRV104 particles were detected in the CIK cells incubated with NH_4_Cl at a concentration of 20 mM (Fig. 2e).

**Fig. 3:**
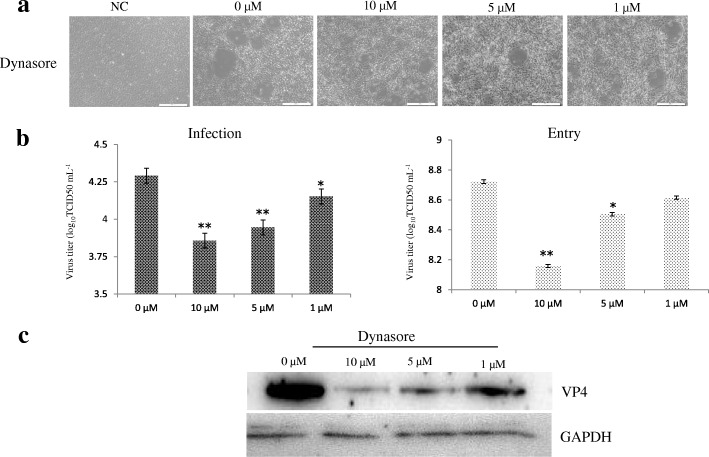
Effect of dynasore on the production of progeny virus. CIK cells were treated with dynasore at the indicated concentrations. **a** CPE of GCRV104 infection viewed under a visible light phase microscope. **b** Rt-PCR assay of virus yield in the supernatants (infection) and CIK cells (entry). (unpaired t-test, * *P* < 0.05 and ** *P* < 0.01). **c** Western blot analysis to monitor viral replication level in CIK cells

As shown in Fig. 3, GCRV104 takes advantage of dynamin-dependent endocytic pathways to infect CIK cells. The CPE of cells treated with various concentrations of dynasore at 5 d post-infection is shown in Fig. 3. RT-PCR assays support that treatment with dynasore significantly inhibits GCRV104 infection and entry (Fig. 3b). Furthermore, western blot results indicate that viral proteins were reduced in GCRV104 infected dynasore-treated cells (Fig. 3c).

### Inhibitor screening for GCRV104 infection

**Fig. 4:**
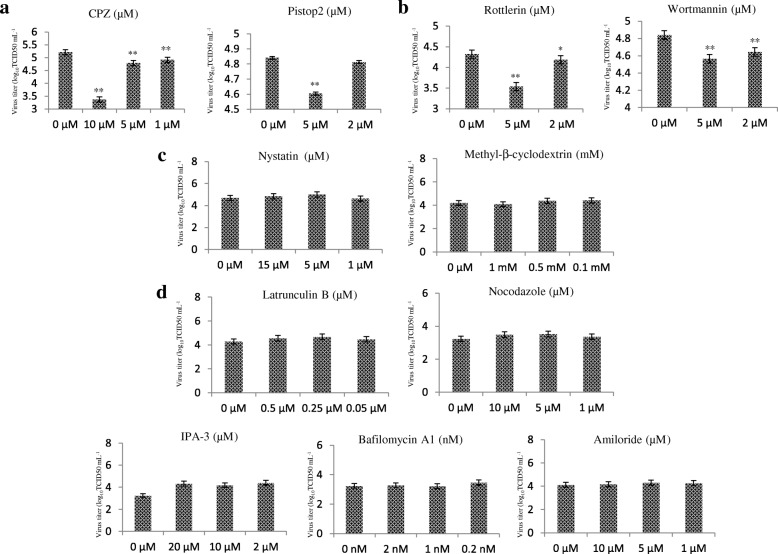
Effect of inhibitors on the production of progeny virus. CIK cells were treated with different inhibitors at the indicated concentrations and then infected with GCRV104 (MOI = 5) for 5 days. Uninternalized virions were removed at 1 hpi. Rt-PCR assay of virus yield in the supernatants. **a** Pistop2 (5 μM and 1 μM) and CPZ (10 μM, 5 μM, and 1 mM) inhibit GCRV104 infection. **b** Rottlerin (5 μM and 2 μM) and wortmannin (5 μM and 2 μM) inhibit GCRV104 infection. **c** and **d** Nystatin (15 μM, 3 μM), Methyl-β-cyclodextrin (1 mM, 0.5 mM, 0.1 mM), Latrunvulin B (0.5 μM, 0.25 μM, 0.05 μM), nocodazole (10 μM, 5 μM, 1 μM), IPA-3 (10 μM, 5 μM, 1 μM), Amiloride (10 μM, 5 μM, 1 μM), and Bafilomycin A1 (2 nM, 1 nM, 0.2 nM) were used for analysis different pathways. Asterisks represent a significant difference from the control (unpaired t-test, * *P* < 0.05 and ** *P* < 0.01)

Several inhibitors were selected to test pathways for GCRV104 infection of host cells. As shown in Fig. 4a, CPZ and pistop2 at specific concentrations all decreased significantly the infectivity percentage of GCRV104 (*P* < 0.01). Both rottlerin and wortmannin reduced GCRV104 infection, compared to non-treated cells (Fig. 4b).

The effects of the cholesterol-sequestering reagent nystatin and the cholesterol-depletion reagent methyl-β-cyclodextrin were used to test whether caveola-dependent endocytosis served as a route for GCRV-104 internalization. Nystatin and methyl-β-cyclodextrin show minimal inhibitory effect of GCRV104 infection (Fig. 4c).

IPA-3, being an allosteric inhibitor of Pak1, was used to define the role of Pak1 in the endocytosis of GCRV-104 [19]. We tested the actin microfilament depolymerization drug Latrunculin B and the microtubules depolymerization drug, nocodazole, to test the roles of cytoskeletal proteins in GCRV-104 infection [25, 26]. Bafilomycin A1 is an inhibitor of the H^+^ ATPase pump [27] and amiloride is an inhibitor of the Na^+^/H^+^ exchanger [28]. In our study, all these inhibitors and thus, their corresponding pathways, had not effect on GCRV104 infection (Fig. 4d).

### GCRV104 entry is clathrin-dependent

**Fig. 5:**
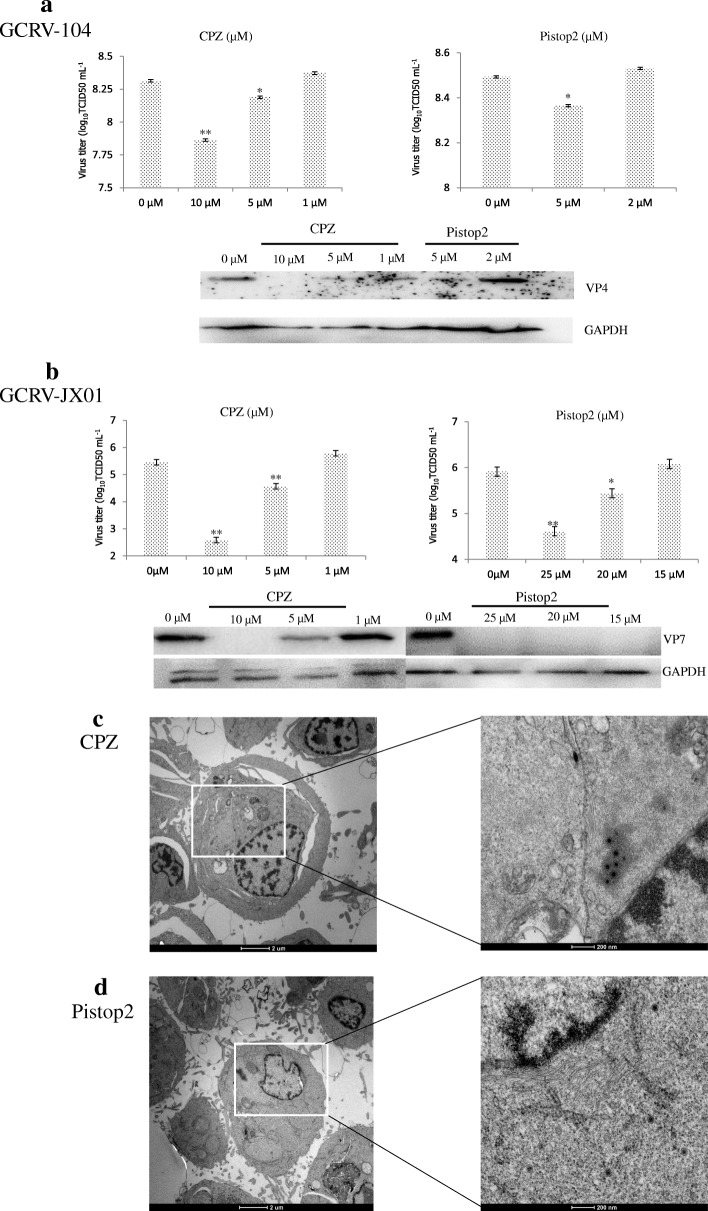
Pistop2 and CPZ inhibit GCRV104 and GCRV JX01 infection. CIK cells were treated with Pistop2 and CPZ at the indicated concentrations. **a** and **b** Rt-PCR assay of virus yield in the supernatants (infection group) and CIK cells (entry group). (unpaired t-test, * *P* < 0.05 and ** *P* < 0.01). Western blot analysis to monitor viral replication level in CIK cells. **c** and **d** Cells were pretreated with indicated inhibitors (**c**:CPZ 10 mM, and **d**: pitstop 2 5 μM) for 1 h at 4 °C, then adsorbed with virus (MOI = 50) for 30 min at 4 °C. The cultures were quickly warmed to 28 °C to start infection, and uninternalized virions were removed at 0.5 hpi. After PBS washes, cells were treated with inhibitors again for 48 h at 28 °C. The processed cells were then fixed in electron microscopy grade fixative at 4 °C

To further determine the effects of CPZ and pistop2 in both entry and replication stages of GCRV104 infection, infected cells treated with CPZ and pistiop2 before and after virus adsorption were collected to quantitate virus copies during the early stage of infection. As expected, a reduction of GCRV104 infectivity was found following CPZ and pistop2 treatment, and the expression level of the viral protein GCRV104-vp4 also diminished (Fig. 5a). In addition, we examined GCRV JX01 following CPZ and pistop2 treatments in the same conditions as GCRV104. Inhibition of viral replication, infection, and protein expression of GCRV JX01- vp5 was also detected at the early stage (Fig. 5b). Finally, we suggest that GCRV104 enters host cells via clathrin- mediated endocytosis, in the same manner as GCRV JX01. As indicated in Fig. 5c and d, treatments with CPZ (Fig. 5c) or pistop2 (Fig. 5d) inhibited GCRV104 entry into CIK cells.

### PI3K and PKC are required for GCRV104 entry

**Fig. 6:**
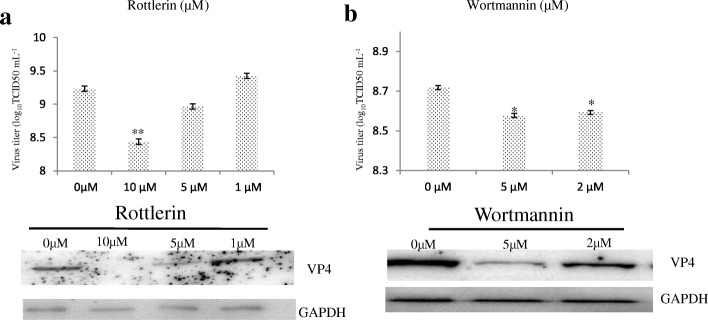
Effect of rottlerin and wortmannin on the production of progeny virus. CIK cells were treated with different inhibitors at the indicated concentrations and then infected with GCRV104 (MOI = 5) for 5 days. Uninternalized virions were removed at 1 hpi. Rt-PCR assay of virus yield in the supernatants. **a** and **b** Rottlerin (10 μM, 5 μM, 1 μM) and wortmannin (5 μM, 2 μM) inhibit GCRV104 infection. Asterisks represent a significant difference from the control (unpaired t-test, * *P* < 0.05 and ** *P* < 0.01). Western blot analysis to monitor viral replication level in CIK cells

Rottlerin and wortmannin both had effects on GCRV104 infection; RT-qPCR and western blot demonstrate that GCRV104 infectivity was strongly decreased in a dose-dependent manner (*P* < 0.01) (Fig. 6). The PI3K inhibitor wortmannin and the PKC inhibitor rottlerin block, at least partially, GCRV104 entry into host cells.

## Discussion

Compared to GCRV-JX01, GCRV104 replication was slower and its CPE was detectable five days post infection (Fig. 1). Figure 2 suggest that a productive GCRV104 entry is pH-dependent. We used dynasore to determine whether dynamin is involved in GCRV104 entry. Our data (Fig. 3) revealed that GCRV104 entry and replication is dependent on dynamin. Of the endocytic pathways used by viruses, the most commonly utilized is the clathrin-mediated endocytic route [29]. Pitstop 2 directly binds to the clathrin terminal domain at a site that overlaps with clathrin box containing accessory protein ligands [30]. CPZ is widely used as an inhibitor of clathrin [31]. We found that GCRV104 entry and infection were significantly inhibited by pitstop2 and Chlorpromazine (Fig. 4a, Fig. 5), while infection was not suppressed by nystatin or methyl-β-cyclodextrin (Fig. 4c). Interestingly, another research group [32] recently demonstrated that GCRV-873 (genotype I) can use caveolae/raft mediated endocytosis as the primary entry pathway to initiate productive infection. These data are not consistent with our previous work [21] on the entry pathway of genotype I virus strain GCRV-JX01. In this study, we tested a range of 2-fold concentrations of pistop2 (0–25 μM) or chlorpromazine (0–10 μM) on GCRV-JX01 or GCRV104 entry. As shown in Fig. 5a, a significant decrease in entry (*P* < 0.01) was observed with concentrations ≥5 μM chlorpromazine, and ≥ 20 μM pistop2. These results are consistent with our previous reports [21]. Similar data were obtained by using the same inhibitors to test GCRV104 (Fig. 4a, Fig. 5 A and C). It is noteworthy, however, that GCRV-JX01 was not strongly suppressed by 15 μM pistop2, while pitsop2 caused a significant decrease in GCRV104 entry (*P* < 0.05) at a concentration of 5 μM. Many reoviruses have been reported to utilize multiple endocytic pathways to enter cells. For example, reoviruses can use both dynamin-dependent and dynamin-independent endocytic pathways to enter cells [33]. Although GCRV-JX01 and GCRV873 both belong to the genotype I group based on bioinformatics analyses [10], these two viruses also show distinct biological features both in vivo and in vitro [7, 32]. Our data indicates that pitstop2 and chlorpromazine block GCRV104 and GCRV-JX01 entry by inhibiting the clathrin-mediated endocytosis pathway.

As shown in Fig. 4b and Fig. 6, we demonstrated that the PKC inhibitor rottlerin blocked GCRV104 entry. Furthermore, pre-treatment with PI3k inhibitor wortmannin also suppressed GCRV104 entry and replication (Fig. 4b and Fig. 6). Wortmannin [19] is a fungal toxin that at low concentrations specifically inhibits PI3-kinase. Mercer et al. [19] has reported that viruses belonging to vaccinia, adeno, picorna, and other virus families take advantage of macropinocytosis. Recently, similar observations have been reported in Ebola virus and spring viraemia of carp virus (SVC), although Ebola virus and SVC enter host cells by both macropinocytosis and clathrin-mediated endocytosis [34]. We investigated the Pak1 inhibitor IPA-3, NHE inhibitor amilorid, vacuolar ATPase inhibitor bafilomycin A1, microtubules inhibitor nocodazole, and actin microfilaments inhibitor Latrunculin B, and all these agents failed to suppress GCRV104 infection (Fig. 4d).

Based on previous reports [21, 32–34], GCRV particles uptake occur through various pathways and depend on many host factors. However, the evidence presented here indicates that GCRV104 entry into CIK cells utilizes predominantly a pH-dependent, clathrin-mediated endocytic or macropinocytosis pathway, and is dependent on dynamin. Our findings provide new insight in understanding the mechanism of GCRV internalization and will be helpful for the development of anti-GCRV drugs.

## Abbreviations

dsRNA: double-stranded RNA
GCRV: grass carp reovirus
hpi: hour post infection
mpi: minute post infection
MRV: mammalian orthoreovirus
